# The electrocortical activity of elite Rubik’s cube athletes while solving the cube

**DOI:** 10.1007/s00221-025-07104-w

**Published:** 2025-05-26

**Authors:** Ali Asghar Zarei, Casper Ravn Frederiksen, Mathias Bundgaard Jensen, Anderson Souza Oliveira

**Affiliations:** 1 Department of Health Science and Technology, Aalborg University, Aalborg, Denmark; 2 Department of Material and Production, Aalborg University, Fibigerstraede 16, building 4, Aalborg Øst, DK-9220 Denmark

**Keywords:** EEG, Rubik’s cube, Spatial memory, Motor learning, Visuospatial memory

## Abstract

Solving the Rubik’s Cube (RC) swiftly demands intricate cognitive abilities to generate strategic and precise movements, and the electrocortical demands in high-level RC athletes have not been explored. Therefore, we aimed at examining the electrocortical activity associated with planning and executing the RC, alongside tasks assessing planning, fine motor skills, spatial working memory, and visuospatial ability. Thirteen experienced male speed-cubers underwent EEG recordings while performing RC-related tasks (planning and execution), Tower of London (TOL), Judgment of Line Angle and Position-15 (JLAP), Memory Match (MEM), and Fine Motor Skills (FMS). Our results demonstrated that speed-cubers presented similar EEG power spectrum when planning and executing the RC across all frequency bands (*p* > 0.05). Pearson’s correlation demonstrated that Delta-band EEG power spectrum in the occipital lobe exhibited a significant association with RC execution (*r* = 0.71, *p* = 0.009), underscoring the importance of visuomotor integration. Similarly, JLAP performance correlated significantly with frontal (*r*=-0.65, *p* = 0.022) and occipital EEG power spectrum (*r*=-0.57, *p* = 0.048) at the Delta-band, emphasizing the role of visuospatial abilities. Moreover, TOL performance correlated significantly with temporal EEG power spectrum at the Delta- (*r*=-0.64, *p* = 0.025) and Theta-band (*r* = 0.67, *p* = 0.011), highlighting the role of planning abilities while solving the RC. In conclusion, this study sheds light on the complex neural mechanisms underlying speed-cubing, revealing intricate neural signatures across multiple brain regions associated with RC-related tasks and isolated cognitive activities. Understanding these neurocognitive underpinnings could pave the way for enhanced training protocols in tasks demanding high-level cognitive and motor skills.

## Introduction

Solving puzzles and games, such as the Rubik’s Cube (RC), engages multiple high-level cognitive processes, including visuospatial reasoning, working memory, procedural learning, and executive planning (Duncan et al. 1996; Unterrainer and Owen 2006; Oberauer and Lewandowsky 2019). The RC, a 3 × 3 × 3 color-matching puzzle, is particularly demanding due to its combinatorial complexity (Korf 1997), requiring solvers to mentally simulate sequences of moves while optimizing for speed and efficiency. This dynamic interplay of cognitive and motor skills has made RC-solving a subject of interest in cognitive neuroscience, as it mirrors real-world tasks requiring rapid decision-making and visuomotor integration (Meinz et al. 2023). Competitive speed-cubing further amplifies these demands, with elite athletes solving the cube in under 5.5 s (GoCube 2021). Cognitive abilities to solve the RC can be used as predictors of real-world outcomes that involve complex skills (Kanfer and Ackerman 1989). Problem-solving tasks such as speed-cubing demand strategic perspective as a behavior directed towards a goal, which often involves setting sub-goals that enable the use of conscious actions (Huang et al. 2017; Weis and Wiese 2019) (Anderson 2015). Therefore, understanding the neural correlates of such expertise can elucidate broader principles of skill acquisition and cognitive plasticity, with potential applications in training protocols for fields requiring high-speed problem-solving, such as medical procedures (e.g., surgery) and aviation.

Recent research has shown that improvements in solving the RC are more associated with fluid intelligence (e.g., the ability to solve novel problems) than working memory capacity, which is the ability to simultaneously store and process information (Meinz et al. 2023). Thus, it may be assumed that different brain regions must be involved in solving the RC. It is plausible to suggest that active planning and working memory, visuospatial ability and fine motor control are required to complete the speed-cubing task, since it is necessary to create a sequence of moves that involve multi-dimensional evaluation throughout the task. The abovementioned cognitive capabilities have been evaluated in isolation using specific experimental paradigms. The first is the tower of London (TOL), which involves re-distribution of colored rings to conform to a pre-established sequence (Unterrainer and Owen 2006). The TOL accesses planning abilities, demanding high engagement of the frontal and pre-frontal cortex (Owen et al. 1996). Research using similar tasks revealed increased theta (Domic-Siede et al. 2021) and beta activity (20 Hz) (Cannon et al. 2014) while performing the TOL. The second is the judgment of Line Angle and Position-15 (JLAP), which consists of assessing the angle and position of a series of 15 lines that form a half-circle starting from the same origin on a plane (Campbell and Collaer 2009; Holden and Hampson 2014). The JLAP assesses visuospatial ability for identification and manipulation of objects, demanding engagement of the pre-frontal, frontal and visual cortices. Superior visuospatial ability is correlated with increased parietal alpha/theta ratio at rest (Eichelberger et al. 2017) and increased overall alpha desynchronization in the parietal area (Gevins and Smith 2000). The third task is memory matching (MEM), which involves memorizing the spatial location of a color pattern and seeking a matching pattern amongst multiple other spatial choices. The task demands generalized cortical activation over multiple regions at the theta and alpha bands (Liu et al. 2018; Marshall et al. 2018). Lastly, speed cubing requires fine motor skills (Floyer-Lea and Matthews 2004), which demands engagement of the premotor and motor cortices, as well as the supplementary motor area (Amunts et al. 1997) and somatosensory cortex (Kilavik et al. 2013). During FMS tasks, there is a reduction in alpha and beta power (Zaepffel et al. 2013; Kilavik et al. 2013; Rueda-Delgado et al. 2019; Espenhahn et al. 2019; Dissanayake et al. 2022) and an increase in gamma power (Babiloni et al. 2016). During speed-cubing competitions, athletes have 15 s to study the state of the cube, examining every face and the arrangement of the colors. Therefore, athletes generate a spatiotemporal strategy to solving the cube prior to start to move it, which requires high cognitive capabilities.

Assessing brain activity is possible through different techniques such as magnetic resonance imaging, functional near-infrared spectroscopy and magnetoencephalography among others (Auriat et al. 2015; Hallett et al. 2021). Among those, surface electroencephalography (EEG) is one of the most popular methods due to its relatively low cost and high temporal resolution, although it has limitations regarding spatial resolution (Auriat et al. 2015; Oliveira et al. 2017). Portability and high temporal resolution may be valuable features for assessing brain activation during speed cubing, as this task demands natural posture and freedom to move. However, to our knowledge no study has explored how electrocortical activity in different brain regions is modulated during the planning and the execution of speed-cubing.

In this study, we evaluated the performance and electrocortical dynamics of speed-cubing athletes while performing speed-cubing simulating a competition, where the athletes have 15 s to establish a solving strategy and subsequently apply it to solve the cube as fast as possible. Moreover, we evaluated the performance and electrocortical dynamics of such athletes while performing tasks that independently assess cognitive capacities related to speed-cubing (planning, fine motor control, spatial working memory and visuospatial ability). Therefore, our study aimed to describe the electrocortical activity of elite speed-cubing athletes during both the planning and execution of speed-cubing. In addition, we established the association between the electrocortical activity from tasks that stimulate specific cognitive capacities in isolation to the activity from planning and solving the RC.

## Methods

### Participants

Thirteen healthy young male adults (23 ± 5 years) with 5.8 ± 2 years of experience in speed-cubing volunteered to participate in this study. The inclusion criteria to participate in the study was a minimum age of 16 years and previous participation in speed-cubing competition. An exclusion criterion was the ingestion of alcohol 24 h before testing. Eleven out of the 13 participants were recruited during their participation in a national speed-cubing tournament in Copenhagen, organized by the World Cube Association in 2019. The average best time from the sample for speed-cubing was 17 ± 5 s. Participants were informed about the experimental procedure and provided verbal and written informed consent to participate in this study. The procedures applied in this study were in accordance with the ethical committee of Northern Jutland practices.

### Experimental design

In a single session, participants were tested in a quiet room while performing speed-cubing and other tasks related to cognitive capabilities (TOL, JLAP, MEM and FMS). The room was set with closed doors and windows to minimize potential visual and auditory distractions to participants while performing the tasks. During the experiments, there were only two researchers and the participant inside the room and researchers remained completely silent throughout task execution. Prior to performing the tests, participants were fitted with a cap containing EEG electrodes for the recordings of electrocortical activity. Initially, a baseline recording of 30 s was performed with the subject seated approximately 1 m from a computer screen displaying a fixation cross at the participant’s sight height. The electrocortical activation of different brain regions was accessed during tasks that individually present similar demands to attempting to solve the RC. Before each task, participants were fully instructed and familiarized prior to recordings, and the order for performing the tasks was randomized. The tasks were the following:

#### Tower of London (TOL)

**Fig. 1:**
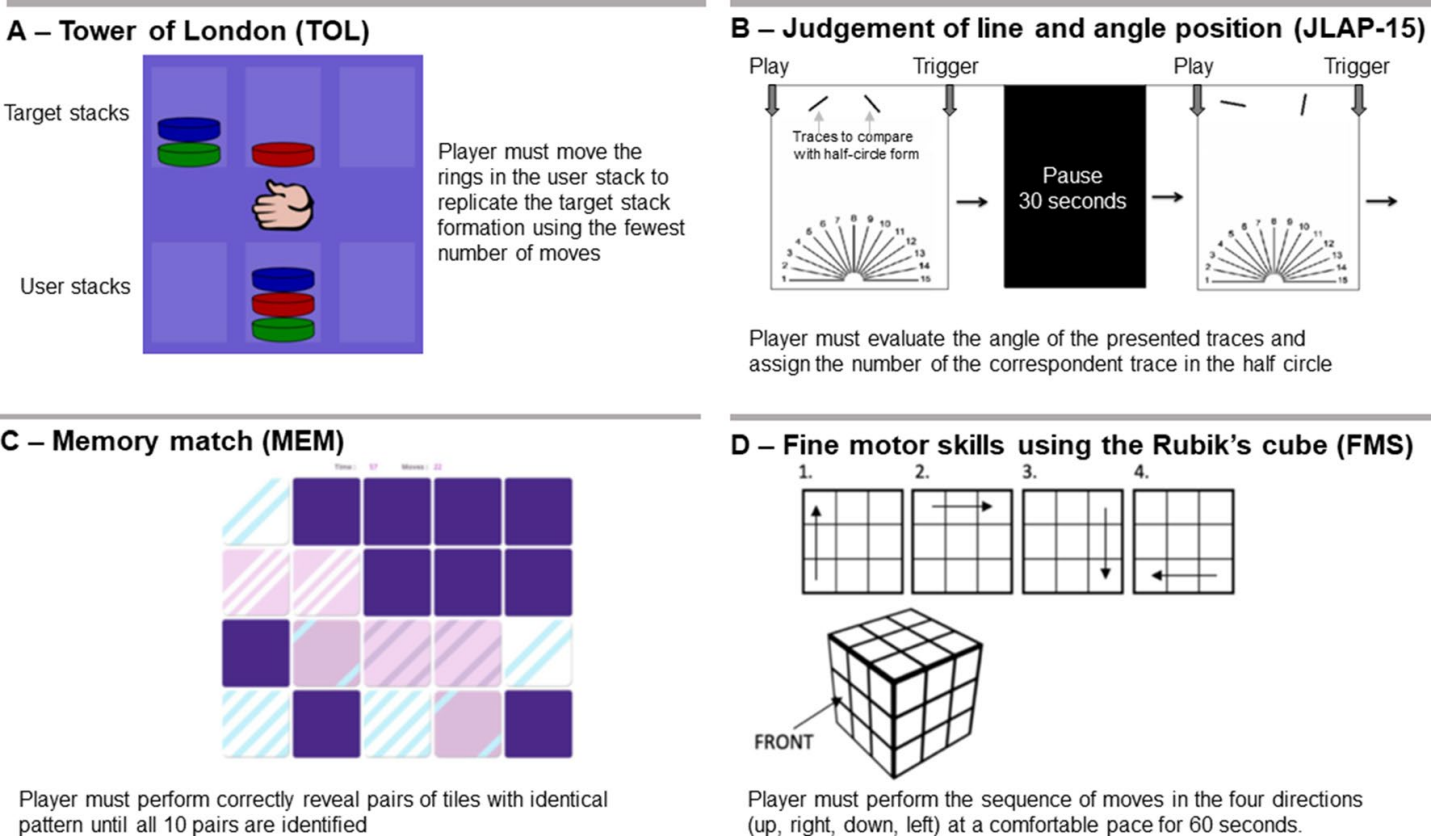
Illustration of the four different tasks that comprised the main cognitive capabilities involved in solving a Rubik’s cube

The task consists of 3 or more colored rings that are distributed across three columns (Unterrainer and Owen 2006). A model of the ring’s organization is shown to the player, and the player must move rings from their starting position to the one demonstrated on the template with as few moves as possible (Fig. 1.A). The software/webpage brainturk.com was used to perform the task. Participants were asked to perform this task 15 times. For the Tower of London task, performance was evaluated using a combined metric, considering the ratio of excessive moves to the minimal moves required, alongside the time taken per task. These values were derived from careful visual analysis of the Tower of London task videos.

#### JLAP

The JLAP test consists of assessing the angle and position of a series of 15 lines that form a half-circle starting from the same origin on a plane (Fig. 1.B) (Campbell and Collaer 2009; Holden and Hampson 2014). Participants were asked to perform this task 10 times.

#### Memory match (MEM)

An online memory match game (Memozor 2021) consisting of a 4 × 5 grid of tiles presented on a 10-inch tablet. There were 10 pairs of tiles presenting identical color patterns, but they were initially hidden (flipped on their backs). Participant was asked to reveal the pattern of pairs of tiles in one turn, if the patterns match, the tiles would be permanently revealed (Fig. 1.C). Otherwise, the two selected tiles would be hidden again, and the participant could select two other tiles. The task was repeated until all 10 pairs of tiles were successfully matched, and the outcome measures were the time and number of moves used to complete the task.

#### Cube manipulation (FMS)

Participants were asked to perform a series of pre-established movements of the cube. After fixing one face as the front of the cube, participants were asked to move the left column up, the upper row to the right, the right column down and the bottom row to the left (see Fig. 1.D), repeating this sequence at a comfortable pace for 60 s. The number of turns performed in 60 s was the outcome measure extracted for each participant. It is noteworthy that there were no signs of physical/mental fatigue during the experiment up to the Rubik’s cube execution. Moreover, participants were offered rest intervals prior to each task, especially prior to starting the Rubik’s cube task, minimizing any possible influence of previous tasks in Rubik’s cube planning and execution.

### Solving the Rubik’s cube

The final task in the protocol was to solve the RC from a random initial configuration. The study participants were presented to the cube and were instructed to firstly study the state of the cube and determine their strategy for 15 s. Immediately following the 15 s to determine the solving strategy, participants started to solve the cube. Electrocortical activity was recorded from both the 15-s solving strategy as RC planning (RC-P) and the RC execution (RC-E). The speed cubing performance was quantified by calculating the average time taken by all subjects in their initial three trials.

### Electroencephalographic and head acceleration recordings

Electroencephalography data were recorded using a wireless 32-channel EEG system (LiveAmp, Brain Products Inc., Gilching, Germany). To minimize the influence of head motion on the EEG recordings, we used an EEG system with active electrodes, fixated the EEG cables with straps to minimize their motion, and also placed a stretchable mesh cap that assisted in maintaining the electrodes with the decided contact to the scalp across the recording. EEG data were recorded with the sampling rate of 500 Hz at all channels (Fp1, Fp2, AFz, F3, F4, Fz, FC1, FC2, FC3, FC4 FC5, FC6, FCz, C1, C2, C3, C4, C5, C6, Cz, CP1, CP2, CP3, CP4, CP5, CP6, CPz, P3, P4, Pz, PO3, PO4). The reference electrode was located on the left mastoid. This EEG system is a gel-based system with active electrodes. The electrode impedances were kept < 20 kΩ during the experiment.

### EEG data analysis

EEG signals were analyzed with BrainVision Analyzer (Version 2.2.2, Brain Products, GmbH). The continuous EEG data recorded from each condition band-passed filtered within 0.5 to 45 Hz using an 8th order FIR filter. Next, blink artifacts and eye movements on the filtered data were corrected using the “Ocular correction independent component analysis” function in BrainVision Analyzer software using independent component analysis (ICA). Subsequently, the time interval of 300 ms around the data point with amplitude exceeding ± 100 µV was marked for rejection to eliminate any remaining non-physiological artifacts (e.g., eye blinks, muscle activity, cable movements) following epoching. The continuous EEG data were then re-referenced to the average reference. Data were then visually inspected, and the remaining artifacts were identified and manually indexed for rejection. Following data cleaning, the continuous artifact-free data were segmented into epochs of 2s duration with 200ms overlap which served as the basis for all following analyses. The number of artifact-free epochs per participant for each condition were as follow: Baseline: 16 ± 1; JLAP: 16 ± 8; FMS: 31 ± 2; TOL: 179 ± 7; MEM: 32 ± 13; RC-P: 17 ± 4; RC-E: 28 ± 8 epochs.

Channel level artifact-free epochs were source localized to the cortex using the built-in low-resolution electromagnetic tomography (LORETA) method of the BrainVision analyzer. Although several studies have used LORETA to estimate electrophysiological activity and reported it as a promising tool in different clinical fields (Zarei et al. 2022; Jadidi et al. 2023), using High-density EEG recording plays an important role in precisely estimating cortical source activity from small brain regions. In line with this, while EEG data were recorded by 32 EEG channels in this study, we consider defining more broad regions of interest as different lobes, including the Frontal, Occipital, Parietal, and Temporal lobes. For each epoch, the current density averaged from all the voxels within a region of interest defined the time-series activity corresponding to the cortical level regions.

The power spectrum from EEG channels was calculated separately for different conditions using a Fast Fourier Transform for each region of interest and the averaged power across epochs were utilized for calculating the mean power density. The power spectrum density at each frequency band (theta, 4–8 Hz; alpha, 8–13 Hz; beta, 14–30 Hz) is defined by the average of the frequency activities among the specified frequency band. The measured power spectrum density at each frequency band, regions of interest, and conditions were then exported for statistical analysis.

### Statistical analysis

All the statistical tests were conducted in RStudio (R version 4.1.3 and RStudio version 2022.02.0). To minimize and eliminate the effect of subjective psychological difference, data in all the conditions were normalized to the baseline condition (the difference between, TOL, JLAP, MEM, FMS, RC-P and RC-E conditions and the Baseline). The Shapiro-Wilk test was used to examine the normality of the data distribution. In case the data followed the normal distribution (from the Shapiro-Wilk test), a one-way repeated measure ANOVA was employed to investigate the brain cortical oscillations between different conditions (TOL, JLAP, MEM, FMS, RC-P, and RC-E) as the within-subject factor at each frequency band and region of interest. However, if the data did not follow the normal distribution, the Kruskal Wallis test was applied as the one-way ANOVA-based non-parametric test. The multiple comparisons problem for all tests was addressed by Bonferroni correction and the level of significance was kept as *p* < 0.05. The association of the power spectrum density for all possible pair-wise comparisons was calculated using Pearson’s correlation analysis for all brain regions at the Delta, Theta, Alpha and Beta bands. Moreover, the association between the RC-E, JLAP and TOL task performances (e.g. time to complete the tasks) and the respective power spectrum density for the task at the different brain regions at the Delta, Theta, Alpha and Beta bands were calculated using Pearson’s correlation analysis.

## Results

### Association between different conditions (brain areas)

**Fig. 2:**
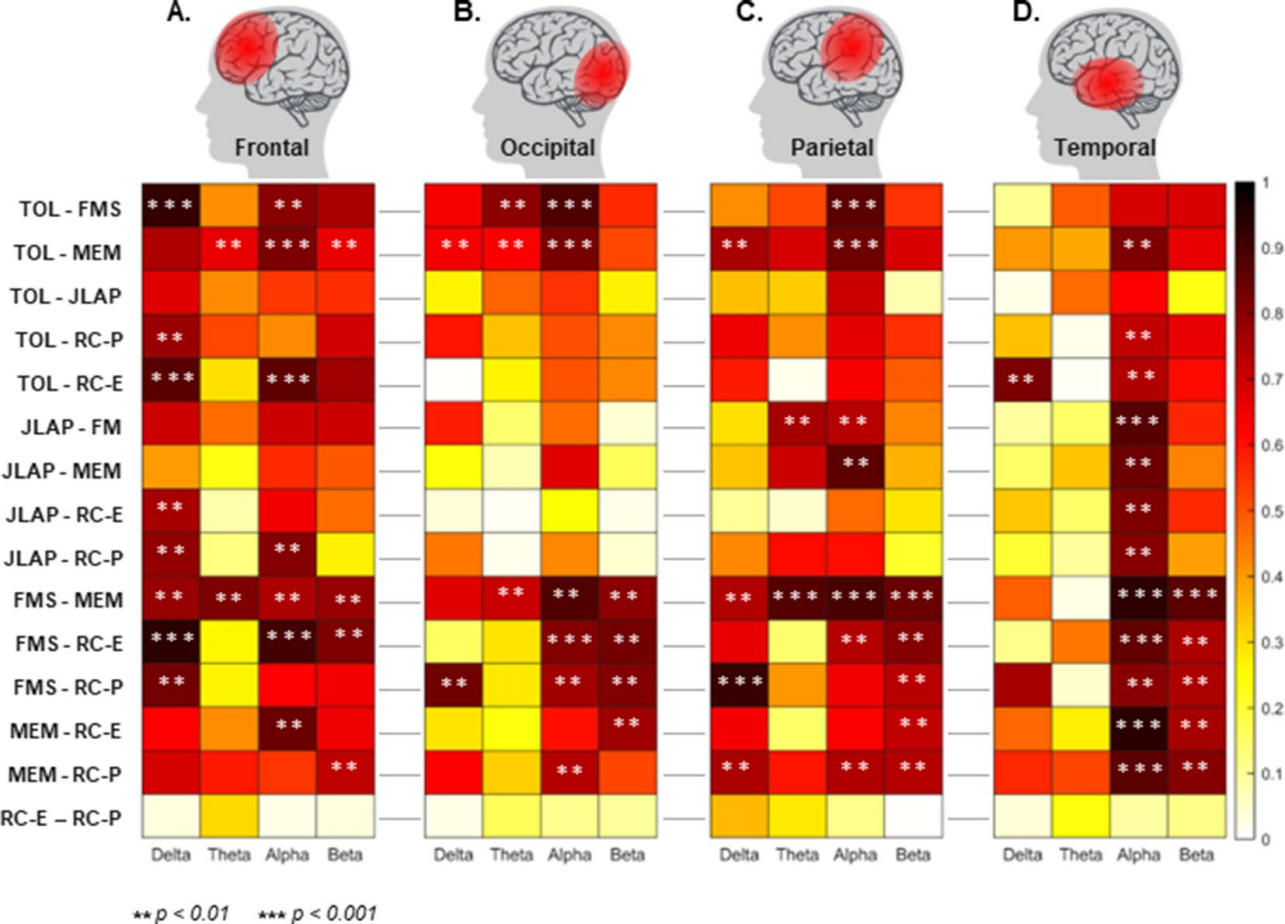
Pairwise correlation of all tasks across four different frequency bands (i.e., Delta, Theta, Alpha, and Beta) and across the Frontal, Occipital, Parietal, and Temporal lobes. The color scale depicts the range of Pearson correlation coefficients for each pairwise comparison. Two different levels of significance are marked with star symbols

Figure 2 shows the pair-wise correlation of all tasks at Delta, Theta, Alpha, and Beta frequency bands and over the Frontal, Occipital, Parietal, and Temporal lobes. There were moderate-to-strong correlations at the frontal lobe between tasks involved in RC (i.e., RC-P and RC-E) and all four isolated cognitive tasks (i.e., TOL, FMS, JLAP, and MEM), mostly at Delta and Beta bands (Fig. 2A, *p* < 0.0125, Bonferroni corrected). Regarding the occipital lobe (Fig. 2B), there were correlations between tasks involved in RC vs. FMS and MEM tasks in the Alpha and Beta bands (*p* < 0.01). The Parietal lobe revealed significant correlations between tasks involved in RC vs. FMS and MEM tasks in the Beta band. Moreover, planning the RC (RC-P) strongly correlated with FMS and MEM in the Delta band. (Fig. 2C, *p* < 0.01). Additionally, the correlation between FMS vs. RC-E and MEM vs. RC-P was found to be statistically significant in the Alpha band. Regarding the Temporal lobe, there were strong correlations between tasks involved in RC and all four isolated cognitive tasks in the Alpha band (Fig. 2D, *p* < 0.01). Moreover, FMS and MEM presented a strong correlation with RCs tasks in the Beta band. Finally, there was a significant correlation at different frequency bands for pair-wise comparisons such as TOL vs. FMS, TOL vs. MEM, FMS vs. MEM, and JLAP vs. MEM.

### Task-related differences in electrocortical dynamics (EEG power normalized to resting EEG)

**Fig. 3:**
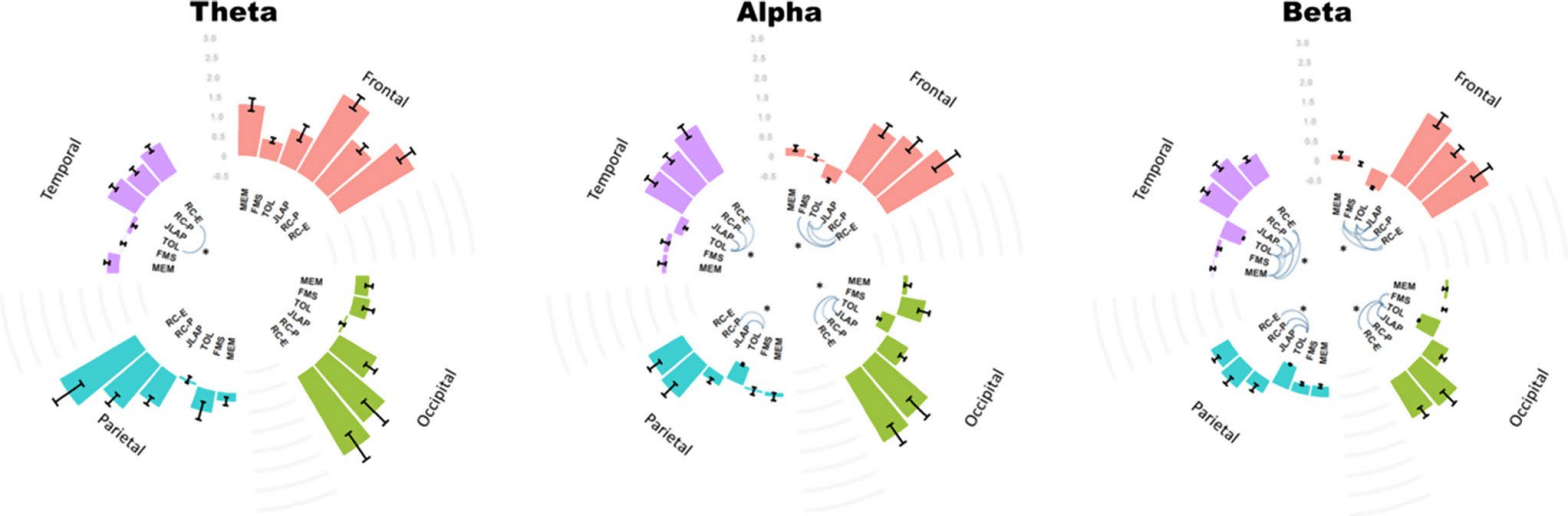
Brain oscillatory activity across theta (**A**), alpha (**B**), and beta (**C**) bands in the frontal, temporal, parietal, and occipital lobes during six tasks. Significant correlations (Bonferroni corrected, *p* < 0.001) are represented by dashed lines connecting the tasks

The theta power from RC-E was generally significantly greater when compared to TOL in the Temporal lobe (Fig. 3A, Bonferroni corrected, *p* < 0.001). In the Alpha band, the oscillatory activity from RC-E and RC-P, and JLAP were significantly higher when compared to TOL across all brain lobes (Fig. 3B). This same pattern was found for the comparisons in the Beta band (Fig. 3C), while the differences between tasks were marginally reduced at the beta band in both the parietal and occipital lobes. No differences between RC-E and RC-P were found across all EEG frequency bands and brain lobes.

### Association between task performance and brain activity

**Fig. 4:**
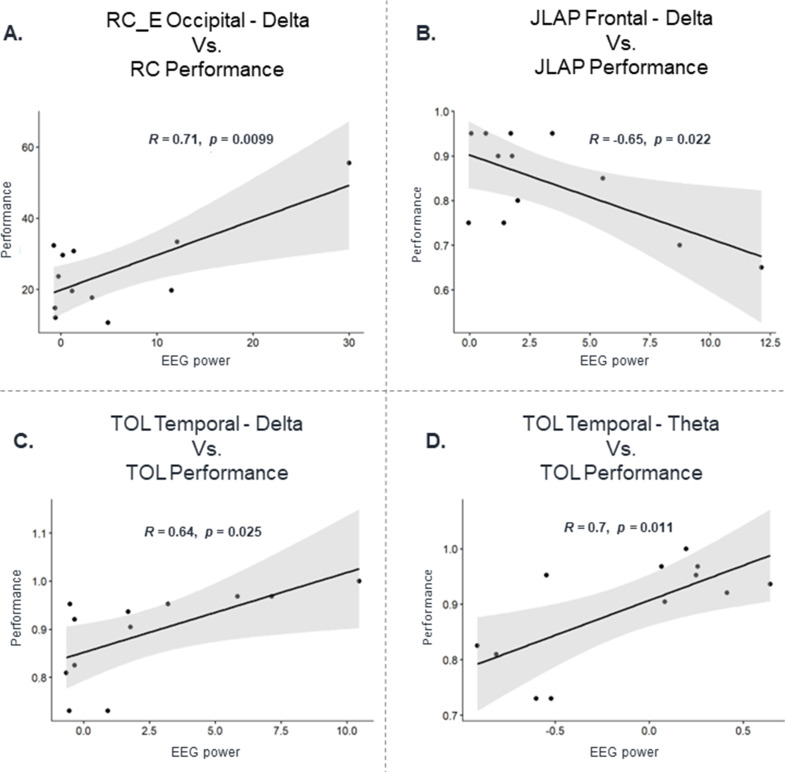
Association between the performance of the tasks and brain oscillatory activity at frequency bands and brain regions demonstrating significant correlations. *R* = Pearson correlation coefficient; *p* = alpha level

Analyzing the performance of the tasks and brain oscillatory activity at four different frequency bands and four brain regions revealed that the performance of speed-cubing is significantly associated with the Delta band activity at the Occipital lobe during RC execution (*r* = 0.71, *p* = 0.009, Fig. 4A). There was a significant association between Delta band activity in the Frontal (*r* = -0.65, *p* = 0.022, Fig. 4B) and Temporal lobes (*r* = 0.64, *p* = 0.025, Fig. 4C) with respect to the performance of JLAP and TOL, respectively. JLAP performance was also significantly associated with Delta band activity in the Occipital lobe (*r* = -0.57, *p* = 0.048). In addition, Theta band activity at the Temporal lobe was also significantly correlated with the performance of the TOL task (*r* = 0.70, *p* = 0.011, Fig. 4D). No significant associations were found between EEG power spectrum and RC planning (*p* > 0.05).

## Discussion

This study investigated the electrocortical activity of elite speed-cubing athletes while planning and executing speed-cubing. Moreover, we explored the association between the electrocortical activity during planning and execution of speed-cubing and the electrocortical activity generated during specific tasks that stimulate specific cognitive capacities. Our main results demonstrated significant associations between electrocortical activities during both planning and execution of speed-cubing and the four isolated cognitive/motor tasks proposed in the study (TOL, JLAP, FMS and MEM) at the frontal lobe. Moreover, substantial increases in EEG spectral power at all brain regions were evident when either planning or executing the speed-cubing moves, with no evident distinction in brain activity between planning and execution. Finally, we found modestly significant associations between electrocortical dynamics at low frequencies (Delta, Theta) and task performance, demonstrating that electrocortical signals can be relevant to quantify high-level skills.

### Electrocortical dynamics in specific cognitive tasks

The association between electrocortical activity during planning and execution of the RC in relation to some specific cognitive tasks demonstrates the demand for multiple abilities to execute the task. The **TOL** assesses planning abilities, which are essential to plan the moves during speed-cubing. The alpha and beta oscillatory activity in the frontal, temporal, and occipital lobes in RC-P and RC-E were significantly higher than the same activity in the TOL task, suggesting that solving RC significantly increases the engagement of the mentioned brain areas at the alpha and beta bands. This elevated engagement may reflect the integration of motor planning, spatial reasoning, and sensorimotor coordination required to solve the RC beyond the purely cognitive demands of the TOL task. Previous studies have demonstrated that alpha and beta desynchronization is linked to higher cognitive and motor demands, particularly in tasks requiring visuomotor coordination and fine motor control (Pfurtscheller and Lopes da Silva 1999; Neuper et al. 2006; Zaepffel et al. 2013; Kilavik et al. 2013). Moreover, frontal and parietal beta oscillations have been associated with internal action planning and motor preparation, especially under conditions of increased task complexity (Engel and Fries 2010; Tzagarakis et al. 2010, 2015). Therefore, the stronger alpha and beta activity observed during RC-P and RC-E compared to the TOL task likely reflects the greater neural integration and cognitive load involved in planning and executing speed-cubing movements.

The **JLAP** assesses visuospatial ability for identification and manipulation of objects. It has been reported that performing JLAP demands the engagement of the pre-frontal, frontal, and visual cortices. Additionally, the superior visuospatial ability is correlated with increased parietal alpha/theta ratio at rest (Eichelberger et al. 2017) and increased overall alpha desynchronization in the parietal area (Gevins and Smith 2000). In line with this, our results demonstrated that cortical oscillatory activity in delta and alpha bands in the Frontal lobe and alpha band in the Temporal lobe significantly correlated with the planning and execution of the RC tasks. Our results provide preliminary insights on the use of surface EEG to evaluate a classical visuospatial ability task such as JLAP, with further studies being relevant to compare the electrocortical dynamics during JLAP between regular people with those with high levels of visuospatial abilities.

**MEM**. Along with planning abilities, memorizing the sequence of moves is highly relevant to solving the RC as fast as possible. The MEM task used in our study demands generalized cortical activation over multiple regions at the theta and alpha bands (Liu et al. 2018; Marshall et al. 2018). Interestingly, we found associations in beta bands across the Frontal, Parietal and Temporal lobes between MEM and planning the RC, but not with executing the RC. This phenomenon was also found in the Alpha band over the occipital, parietal, and temporal lobes and the delta band in just the parietal lobe.

Alpha band activity in the parietal lobe relates to attentional control processes (Palva et al. 2005), while the alpha rhythm at parietal and occipital areas is closely related to memory load during retention and visuo-spatial memory tasks (Jensen et al. 2002; Bastiaansen et al. 2002). Our results may indicate the allocation of attentional resources to maximize the retention of information through working memory, in which the effective memorization process relies on visual cues or mental imagery of the sequence of moves. Moreover, the delta band activity in the parietal region may be linked to attentional processes or working memory (Jung et al. 2020), both of which are crucial for effective planning and memorization of the RC movements. Our results may suggest that speed-cubing athletes may employ a strategic approach during the planning phase of the RC task, which is subsequently implemented during the execution phase. However, further studies deepening our understanding on the differences in electrocortical activity between planning and executing the RC are required to confirm our suggestion.

Refined bilateral hand dexterity is crucial to solving the RC as fast as possible. **FMS** engages the premotor and motor cortices, as well as the supplementary motor area (Amunts et al. 1997) and somatosensory cortex (Kilavik et al. 2013). FMS evoke reductions in EEG alpha and beta power (Zaepffel et al. 2013; Kilavik et al. 2013; Rueda-Delgado et al. 2019; Espenhahn et al. 2019; Dissanayake et al. 2022), as well as increases in gamma power (Babiloni et al. 2016). In line with this, our results revealed that alpha and beta band activities of executing the RC (RC-E) were significantly correlated with the FMS task across all brain areas (i.e., parietal, temporal, occipital, and frontal). Moreover, we found a significant correlation between RC-P and FMS in the beta band over the parietal, occipital, and temporal lobes. Such results suggest that the planning stage of the RC task is influenced by the individual’s level of fine motor skill proficiency. We also found delta band activity of RC-P was correlated with FMS task over parietal, frontal, and occipital brain areas. The observed correlations between FMS and EEG band activities across different brain regions provide valuable insights into the neural underpinnings of motor coordination and planning. Understanding these relationships may have implications for developing interventions or training protocols to enhance motor skills and optimize performance in tasks that require precise hand dexterity and coordination (Sidhu and Cooke 2021).

### Planning vs. executing the Rubik’s cube

In our study we did not find associations between RC-P and RC-E. Previous studies have shown that motor imagery is effective in improving real-life motor actions in rehabilitation (Machado et al. 2019), while somatosensory and motor brain areas present remarkably similar activities when imagining or executing hand movements (Miller et al. 2010) or even walking (Stolbkov et al. 2019). Our study corroborates such findings when similar electrocortical activity is reported across all investigated brain regions when planning or solving the Rubik’s cube. Despite similar power spectrum profiles between planning and solving the Rubik’s cube, the activities were not associated regardless of the brain region. It would be expected that not only the general power magnitude could be similar, but also that planning and execution the task would demand similar patterns across participants.

### Association between electrocortical activity and real-world performance

Our results demonstrated that solving the Rubik’s cube, the JLAP and the TOL tasks were significantly associated with the EEG activity in specific brain regions at low-frequency (Delta and Theta) bands. Solving the Rubik’s cube was associated with the occipital EEG power at the delta band, which may be related to the high demands for visuomotor integration throughout the task. The JLAP task relied heavily on visuospatial ability, which requires involvement of the pre-frontal and frontal cortices, justifying the significant association with the JLAP performance. Finally, the TOL performance presented significant association with the EEG power from the temporal lobe at the Theta and Delta bands, demonstrating that planning abilities may be associated with such EEG features. However, noteworthy that our correlations were estimated using a limited number of points (*n* = 13), and further studies including a greater sample size would be necessary to confirm our results.

### Limitations and future work

The first limitation of this study is the relatively small sample size (*n* = 13), which limits the statistical power from analyses of variances and Pearson’s correlations (Button et al. 2013). Secondly, our sample included only young adult males, which restricts the generalizability of the results, as research has shown that both sex and age can influence cognitive performance and EEG signal characteristics (Gur and Gur 2016; Hashemi et al. 2016). Finally, the allowance for head movement during RC execution introduces potential contamination from movement-related artifacts, which can be difficult to fully eliminate in traditional EMG settings (Oliveira et al. 2016, 2017; Richer et al. 2020). Regarding future work, it is relevant to replicate these findings in larger and more diverse cohorts, including females and individuals with varying levels of experience. Longitudinal studies assessing the impact of training on the neural correlates of speed-cubing may provide further insight into the neuroplasticity associated with complex visuomotor skill acquisition. Moreover, incorporating complementary neuroimaging modalities (e.g., fNIRS, MEG) may enhance ecological validity and signal fidelity in future research.

## Conclusion

In summary, our study demonstrated that several brain regions (frontal, parietal, temporal and occipital) present electrocortical activity that is associated with the electrocortical activity generated during planning/executing the Rubik’s cube. There is no detectable difference in the power spectrum generated when planning or solving the Rubik’s cube, demonstrating that the planning phase of the speed-cubing competition establishes a cognitive solving strategy that will be “copied” during the execution phase. Moreover, modest yet significant associations between electrocortical activity and the performance of real-world tasks (RC-E, JLAP and TOL) demonstrates that EEG features may have potential to underpin progress in cognitive learning involving visuomotor and planning abilities.

## Data Availability

No datasets were generated or analysed during the current study.
